# Temporal vs. spatial variation in stress-associated metabolites within a population of climate-sensitive small mammals

**DOI:** 10.1093/conphys/coab024

**Published:** 2021-05-03

**Authors:** Ashley L Whipple, Chris Ray, Max Wasser, James N Kitchens, Alisa A Hove, Johanna Varner, Jennifer L Wilkening

**Affiliations:** 1 Department of Ecology and Evolutionary Biology, University of Colorado, Boulder, CO 80309, USA; 2 Institute of Arctic and Alpine Research, University of Colorado, Boulder, CO 80303, USA; 3 Department of Biology, Warren Wilson College, Asheville, NC 28778, USA; 4 Department of Biological Sciences, Colorado Mesa University, Grand Junction, CO 81501, USA; 5 US Fish and Wildlife Service, Southern Nevada Fish and Wildlife Office, Las Vegas, NV 89130, USA

**Keywords:** American pika, glucocorticoid metabolite, habitat quality, *Ochotona princeps*, stress physiology

## Abstract

Temporal variation in stress might signify changes in an animal’s internal or external environment, while spatial variation in stress might signify variation in the quality of the habitats that individual animals experience. Habitat-induced variations in stress might be easiest to detect in highly territorial animals, and especially in species that do not take advantage of common strategies for modulating habitat-induced stress, such as migration (escape in space) or hibernation (escape in time). Spatial and temporal variation in response to potential stressors has received little study in wild animals, especially at scales appropriate for relating stress to specific habitat characteristics. Here, we use the American pika (*Ochotona princeps*), a territorial small mammal, to investigate stress response within and among territories. For individually territorial animals such as pikas, differences in habitat quality should lead to differences in stress exhibited by territory owners. We indexed stress using stress-associated hormone metabolites in feces collected non-invasively from pika territories every 2 weeks from June to September 2018. We hypothesized that differences in territory quality would lead to spatial differences in mean stress and that seasonal variation in physiology or the physical environment would lead to synchronous variation across territories through time. We used linear mixed-effects models to explore spatiotemporal variation in stress using fixed effects of day-of-year and broad habitat characteristics (elevation, aspect, site), as well as local variation in habitat characteristics hypothesized to affect territory quality for this saxicolous species (talus depth, clast size, available forage types). We found that temporal variation within territories was greater than spatial variation among territories, suggesting that shared seasonal stressors are more influential than differences in individual habitat quality. This approach could be used in other wildlife studies to refine our understanding of habitat quality and its effect on individual stress levels as a driver of population decline.

## Introduction

Reversing the current, unprecedented rate of human-caused biodiversity loss (IUCN 2019) will depend on identifying stressed populations before they disappear and determining how stress is mediated by the environment (Homyack, 2010). The growing field of conservation physiology provides tools for discovering the mechanisms that support biological diversity and that govern individual- and population-level responses to environmental change and stressors (Cooke *et al.*, 2013). Almost half of the work published in the journal ‘Conservation Physiology’ over the past 5 years has focused on stress physiology. In a 2013 review, the majority of 287 stress physiology papers on non-fish vertebrates focused on responses to capture and handling (Baker *et al.*, 2013). Conversely, few studies focused on stress response to changes in land use or environmental influences (Baker *et al.*, 2013), such as habitat quality. Habitat quality is a key factor in determining reproductive fitness and subsequent population growth or decline in numerous species (Davenport *et al.*, 2000, Franklin *et al.*, 2000, Gunnarsson *et al.*, 2005, Johnson, 2007, Holbrook *et al.*, 2019, Navarro-Castilla and Barja, 2019). If low-quality habitats do not provide the energy needed to sustain allostasis (physiological stability in the presence of actual or perceived stressors), then additional energy demands can push an individual into allostatic overload (a cumulative effect of chronic stress) and reduce individual fitness (McEwen and Wingfield, 2003).

Many ephemeral factors (e.g. extreme weather events, exposure to predators, behavioral differences, reproductive status, seasonal variation, etc.) can affect levels of stress (McEwen and Wingfield, 2003, Baker *et al.*, 2013), but a consistent stressor such as poor habitat quality should lead to differences in chronic stress among individuals that are consistent through time (Ellis *et al.*, 2012), particularly for individually territorial animals. If the stress level of each individual in a population varies predictably in response to an environmental stressor, then that response can be used as a metric of population state (Madliger and Love, 2014). However, few studies have quantified seasonal patterns in stress within and among individuals. A study of the capercaillie (*Tetrao urogallus*), a non-migratory grouse, found that sex and individual identity accounted for as much as 37% of variation in stress among free-living individuals, while environmental variables accounted for only 5.1% of the variation (Coppes *et al.*, 2018). By identifying individuals, Coppes *et al.* (2018) detected strong, sex-specific, seasonal variation within individuals, highlighting the need to track stress in known individuals, especially when individuals are non-territorial and share common habitat.

**Figure 1:**
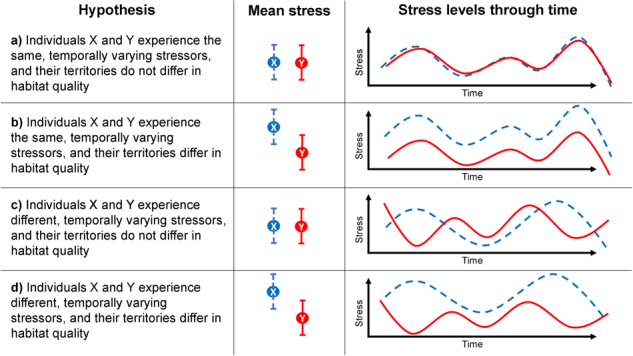
Examples of hypothesized variation in stress for two hypothetical individuals. Temporal variation that is synchronous (a and b) or asynchronous (c and d) is superimposed on potential habitat-mediated differences in mean stress level (central panel). Mean stress levels in territorial animals should be affected by habitat quality. In this example, individual X shows higher stress in (b) and (d), suggesting that it might occupy a territory of lower quality.

Here, we argue that differences in habitat quality should affect differences in mean stress among individuals over time, while individuals occupying similar habitats should exhibit more similar mean stress levels over time. In addition to spatial variation, stress should also vary temporally (Fig. 1). If stress varies synchronously among individuals over time, then climate or seasonal processes might be important covariates of stress (Fig. 1a and b). If stress varies asynchronously among individuals, then each individual is experiencing a different temporal pattern of stressors (Fig. 1c and d). Regardless of temporal variation in stress, differences in mean stress level among individuals might signal long-term differences in exposure to stressors, indicating differences in habitat quality.

Our ability to measure how habitat quality affects stress might also vary with the scale of analysis and with our understanding of what constitutes high-quality habitat. For capercaillie, effects of habitat quality (proportion of open forest) on stress were apparent at very local scales (20-m radius) but not at home-range scales (400-m radius) (Coppes *et al.*, 2018). Canopy cover was patchy within home ranges, so the strength of its effect was difficult to detect when canopy cover was averaged at the larger scale (Coppes *et al.*, 2018). In a study of spotted salamanders (*Ambystoma maculatum*), Homan *et al.*, (2003) failed to find effects of habitat quality on individual stress but also questioned whether the metrics used in their study (canopy cover and soil moisture) were adequate indicators of habitat quality. Both capercaillie and spotted salamanders use different portions of their home range throughout the year and have the potential to move through a gradient of habitat quality. However, such considerations of study scale and habitat metrics should be more straightforward for territorial species with relatively small and permanent territories, as well as for species with well-characterized habitat needs and physiological limits.

We used the American pika (*Ochotona princeps*; hereafter, pika) as a model to explore variation in stress within and among individuals. Pikas are small (~150 g) mammals that live in rocky habitats in western North America, including talus slopes, boulder fields and lava beds (Smith and Weston, 1990, Rodhouse *et al.*, 2010). Pikas are individually territorial and do not migrate after establishing a territory (Smith and Weston, 1990). Pika territories are relatively small (14–34 m diameter; Smith and Weston, 1990) and are generally centered on a winter food cache or ‘haypile’ of herbaceous vegetation harvested during the summer and consumed over the winter (Huntly *et al.*, 1986, Dearing, 1996). Pikas do not migrate or hibernate, so each animal is exposed to the habitat within its territory throughout the year (Krear, 1965). Pika occurrence and persistence appear to be influenced by snowpack, association with sub-surface ice features and climate (Hafner, 1994, Millar and Westfall, 2010, Wilkening *et al.*, 2015, Yandow *et al.*, 2015, Johnston *et al.*, 2019). Experiments have shown that pikas cannot tolerate temperatures >24°C without immediate access to cooler temperatures, which likely explains their affinity for rocky habitats with relatively cool and mesic sub-surface microclimates (MacArthur and Wang, 1974, Smith, 1974a, Hafner, 1993, 1994; Henry *et al.*, 2012; Smith *et al.*, 2016).

Pikas are currently experiencing range retractions (Beever *et al.*, 2016, Jeffress *et al.*, 2017) and local extirpations in many parts of their range (Nichols *et al.*, 2016, Jeffress *et al.*, 2017, Stewart *et al.*, 2017). Climate is an apparent driver of pika losses (Hafner 1994; Grayson 2005; Beever *et al.*, 2003, 2010, 2011; Wilkening *et al.*, 2011, 2015; Stewart *et al.*, 2017; Rodhouse *et al.*, 2018), and several projections suggest dramatic losses during this century (Galbreath *et al.*, 2009, Calkins *et al.*, 2012, Stewart *et al.*, 2015). However, climate vulnerability predictions for pikas in eight national parks (Schwalm *et al.*, 2016) suggest a more complex future in which some populations might persist into the next century, while others are likely to be extirpated. This potential complexity was corroborated by a range-wide analysis showing that the determinants of pika occurrence appear to vary by ecoregion (Smith *et al.*, 2019). Importantly, all studies of pika range dynamics have relied on occurrence data to determine past and present habitat, and none have used metrics of individual stress to refine our understanding of habitat quality. All range projections have predicted pika vulnerability in terms of local extirpation, rather than the potential for population declines that might be reversed (before extirpation) through management interventions informed by an understanding of how habitat quality mediates stress in individuals. This deficit in our understanding of habitat-mediated stress, combined with the complexity and uncertainty of projected pika futures, highlights the need for studies of physiological stress to advance pika conservation. The potential to characterize temporal variation in stress vs. differences in mean stress (Fig. 1) should make such analyses valuable for differentiating purely temporal drivers of stress from those with a spatial component suggestive of habitat effects.

To address questions about the spatiotemporal pattern of stress in pikas, we measured glucocorticoid metabolite (GCM) concentrations collected non-invasively from fecal samples deposited by free-living animals. Glucocorticoids, which aid in the mobilization of response to a stressor, are just one component of the complex stress response in mammals (Moberg 2000; Reeder and Kramer 2005). If an animal cannot reconcile a stressor and return hormones to regular levels, glucocorticoids can become chronically elevated (Boonstra *et al.*, 1998; McEwen and Wingfield 2003). Chronically high glucocorticoid levels can lead to fitness consequences, including decreased body mass, reduced reproduction and increased mortality (Romero *et al.*, 2009; Dantzer *et al.*, 2014). The use of corticosterone enzyme immunoassays to measure fecal GCM concentrations has been biologically validated for pikas (Wilkening *et al.*, 2013). Field studies have found that in pikas confronted with a stressor (trapping and restraint), fecal GCM levels increased (Wilkening *et al.*, 2013), and individuals with higher GCM levels before capture were less likely to survive the following year after capture (Wilkening and Ray 2016). Fecal GCM represents a ~12.5-hour moving average of circulating GC’s (Wilkening *et al.*, 2013), and our sample collection likely will capture multiple defecation events, reducing the effect of circadian rhythms. In previous studies where feces were collected over time from trapped pikas, there was no detection of circadian rhythm in the GCM signal (Wilkening *et al.*, 2013). Here, we use GCM level as an indicator variable for stress and hypothesize that GCM levels should be indicative of territory quality and should be higher where pikas are stressed by occupying habitats of lower quality.

To explore hypotheses related to mean stress level and territory quality (Fig. 1), we used data on GCM concentration measured over time across a number of pika territories to model temporal and spatial variation in stress as a function of broad and local habitat characteristics. We predicted that samples collected from different territories would differ in mean GCM levels, allowing us to model stress as a function of local habitat characteristics. This approach could be used in other studies to refine understanding of habitat quality and its effect on individual stress as a driver of population decline.

## Materials and methods

### Study system

**Figure 2:**
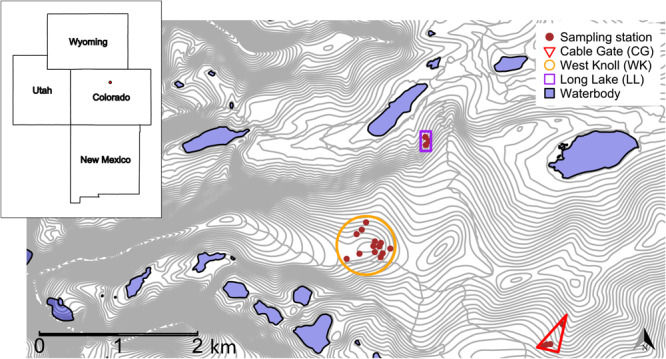
Study site locations on Niwot Ridge, Boulder County, Colorado, USA. Each red dot within a site is a fecal pellet sampling station (*n* = 20) located within a unique territory.

Fecal pellets were collected from pikas living above and below treeline within the Niwot Ridge Long-Term Ecological Research site (40° 3’N 105° 36’W) in Boulder County, Colorado. On Niwot Ridge, pikas occupy taluses that vary in depth (0.5 to 1.5 m) and clast size (10 to 200 cm in longest axis). Fecal pellet sampling stations (hereafter, stations) were established at haypiles separated by at least 50 m, which likely belonged to different individuals occupying distinct territories (Smith and Ivins 1984). We chose one station per territory, near the most conspicuous haypile site within the territory, where the territory owner was likely to deposit pellets repeatedly. Stations were located in 20 territories at varying elevations (3279–3616 m) in three study sites separated by <3 km: Cable Gate (CG), West Knoll (WK) and Long Lake (LL) (Fig. 2). WK (13 stations) was above treeline on a knoll while CG (4 stations) and LL (3 stations) were below treeline.

### Fecal collection method

Pikas produce two types of feces: soft caecal feces are re-ingested and later deposited as firm fecal pellets (Smith and Weston 1990; Nichols 2010). Pellets are known to change color and texture as they age (Nichols 2010), so we collected only ‘fresh’ pellets that were greenish in color and still fibrous inside. In addition, we targeted only large pellets (>2.75 mm in diameter) to avoid sampling from juvenile pikas because GCM levels vary between age classes (Wilkening *et al.*, 2013). Each station was visited every 2 weeks from 8 June through 5 October 2018. During each visit, fresh pellets were collected if available, and older pellets were cleared to ensure that only fresh pellets were collected on subsequent visits. Samples were stored in paper envelopes labeled with sampling date and coordinates. To avoid contamination, we scooped pellets using the envelope flap or pushed pellets into the envelope using a stick or rock. We attempted to collect 10–15 pellets per sample (~0.1 g total) for GCM analyses (Wilkening *et al.*, 2013). Within 24 hours of collection, samples were stored at −20°C.

### Habitat survey

Habitat characteristics within pika territories were recorded using protocols patterned on previous studies (Rodhouse *et al.*, 2010, Erb *et al.*, 2011, Jeffress *et al.*, 2013, Ray *et al.*, 2016). In July 2019, we recorded pika-associated habitat metrics within a 12-m radius of each station. Fine-scale habitat characteristics and land cover types considered predictive of pika occupancy were recorded, including talus depth (depth of deepest crevice, estimated visually from surface rock to ground under talus and categorized as <0.5 m, >0.5 m, >1.0 m or >1.5 m) and clast size (*L_R1_* and *L_R10_*, where *L* = length of longest axis in meters, *R1* = largest rock within 12 m of station and *R10* = 10th-largest rock within 12 m of station). Land cover was categorized as six types (rock, bare, grass, forb, shrub and tree), and percent cover of each type within the 12-m radius was estimated visually using Daubenmire’s (1959) scale.

### Sex determination

Because GCM levels vary between sexes (Wilkening *et al.*, 2013), we split one sample from each territory for genetic analysis to determine the sex of the pika depositing fecal pellets in that territory. DNA was extracted from fecal pellets using the Qiagen DNeasy Fast Stool kit, and DNA concentration (ng/μl) and purity (A_260_/A_280_) were measured using a Nanodrop Lite Spectrophotometer. We used multiplex polymerase chain reaction (PCR) targeting both the male-specific SRY gene and an autosomal microsatellite gene (*Ocp10*) using primer sequences in Table 1 (Lamb *et al.*, 2014). We performed three replicates of the PCR reaction per sample. All PCR reactions were a 17 μl volume containing 11 μl 2× GoTaq Green Master Mix, 2 μl of each 10 μM *SRY* F/R primers, 2 μl of each 10 μM *Ocp10* F/R primers and 2 μl of sample DNA. We included two negative controls lacking pika DNA per PCR run to control for contamination. PCR conditions were as follows: 94°C for 2 min, 40 cycles of 94°C for 30 s, 51°C for 30 s, 72°C for 30 s and a final 5 min extension at 72°C. Samples were then held at 4°C. PCR products were visualized on 2% agarose gels stained with ethidium bromide. Samples were identified to sex using the following criteria: male = *SRY* fragments visible in two of three replicates or *Ocp10* and *SRY* fragments present in the same replicate; female = *Ocp10* fragments were present in at least one of the three replicates, and *SRY* fragments were never present; unknown = only *SRY* fragments were present in one replicate, target genes never amplified or samples were too small to subset for genetic analyses**.**

**Table 1:** Primers used in this study targeting *O. princeps*–specific Y-linked and autosomal regions (adapted from Lamb *et al.*, 2014)

Region	Primer sequence	Size (bp)	Reference
Sex-determining region Y-chromosome (SRY)	F: AATGCATTCATACTATGGTC R: CTCTGTAAGCTTTTTCCACTG	117	Lamb *et al.*, 2014
Autosomal microsatellite (Ocp10)	F: TCCCAGTCACGACGTCCAATTTGGCTGTTA R: GTTTCTTCCAGTGTCTGGCATACGGTAAGC	179–203	Peacock and Kirchoff, unpublished (GQ461705)

### Hormone analysis

GCMs are metabolized stress-associated hormones present in feces that can be used as a measure of glucocorticoids in the body (Keay *et al.*, 2006; Homyack 2010; Dantzer *et al.*, 2014). GCMs in feces represent a time-averaged metric of glucocorticoids in the body (Romero and Reed 2005; Sheriff *et al.*, 2011; Dantzer *et al.*, 2014). For pikas, this time window is ~12.5 hours (Wilkening *et al.*, 2013). GCMs are therefore used as a metric of ‘chronic’ stress (Keay *et al.*, 2006; Dantzer *et al.*, 2014), as opposed to metrics of ‘acute’ stress such as corticosterone in blood (Romero and Reed 2005; Sheriff *et al.*, 2011; Ellis *et al.*, 2012). GCM concentrations were measured in fecal pellets (hereafter, samples) using a commercially available corticosterone enzyme immunoassay (Arbor Assays, Ann Arbor, MI) previously validated for *O. princeps* (Wilkening *et al.*, 2013). We followed the standard kit protocol. Dry samples were weighed to 0.1 ± 0.02 g and 200-proof ethanol was added in proportion to sample mass. After shaking for 30 min, samples were centrifuged and the supernatant was transferred to a new vial. Supernatant was concentrated using a DNA SpeedVac and stored at −20°C until all samples were prepared for the GCM assay. Supernatant was reconstituted to its original volume using a mix of assay buffer and ethanol (<5% of volume) before loading onto enzyme immunoassay plates. Optical densities (450 nm) were read with a Thermo Scientific Multiskan EX Microplate Reader using Ascent Software version 2.6. Samples, standards and controls were assayed in triplicate and the average of each triplicate was used to calculate GCM concentrations. We used seven standards of known corticosterone concentrations (5000, 2500, 1250, 625, 312.50, 156.25 and 78.125 pg/ml) and non-specific binding (NSB) and maximum binding (B_o_) controls. If a sample reading was outside of the range of the NSB or B_o_ averages, then the reading was dropped from analysis. Sample concentrations were calculated using a standard curve and reported as pg GCM/g of dried feces. Inter-assay coefficients of variation were 2.6% and 8.5% for the low- and high-binding controls and 14.7% and 6.3% for low (78.125) and high (5000) standards, respectively. Intra-assay coefficients of variation were between 4.2% and 8.95% for five plates.

### Linear mixed-effects models

Log GCM concentration was used as the response variable in linear mixed-effects models to characterize variation in stress among stations (territories) and within stations over time. We fit a set of models based on up to six predictor variables (Table 2) plus the random effects of station (to account for repeated measures at each station) and date (to account for effects of weather). Our sampling scheme could warrant stations to be nested within site; however, with only three sites, we could not reliably estimate the random variance introduced by site. Instead, we included site as a predictor variable in our candidate models. Predictor variables were selected to account for effects of lab procedures (lab), inter-individual differences (sex), temporal trends, broad-scale habitat characteristics and fine-scale habitat characteristics (Table 2). A fixed effect of enzyme immunoassay plate was considered to account for any effects of laboratory procedures that might affect samples analysed on the same plate. Pika sex was categorized as male, female or unknown. Fixed effects of day of year (DOY, Julian date) and DOY^2^ were included to characterize both a linear trend and any seasonal peak or trough (nonlinearity) in GCM concentration. We used broad-scale habitat characteristics to model spatial effects on GCM concentrations that might affect nearby territories, including fixed effects of elevation, aspect (cosine transformed) and site. Elevation was mean centered and divided by standard deviation. Though not directly measured, site-level differences such as site size and density were captured in the fixed effect of site. A subset of models included fixed effects representing fine scale habitat differences between territories, such as talus depth, rock size, mean rock size (0.5[*L_R1_* + *L_R10_*,]), rock size variation ([*L_R1_*—*L_R10_*]/[0.5*(L_R1_* + *L_R10_*)]) and vegetative qualities that could affect diet (graminoid to forb ratio, percent forb cover and the sum of percent graminoid, forb and shrub cover) (Table 2). Talus depth (coded as a categorical variable) was explored because talus may act as an insulator from surface conditions, and deeper talus may provide a better refuge during extreme heat (Moyer-Horner *et al.*, 2015). Rock characteristics were explored because we lack prior knowledge of how structural complexity of the habitat might affect habitat quality. Lastly, interactions between DOY and elevation, DOY and site, DOY^2^ and elevation and DOY^2^ and site were explored because we expected the timing of maximum and/or minimum GCM concentration to be affected by elevation or site qualities. Correlation among predictor variables was calculated using Kendall’s tau (to accommodate ordinal variables), and variables with high correlation (|r| > 0.7) were not included in the same model; instead, each pair of correlated predictors was included in a separate, parallel set of candidate models.

**Table 2:** Candidate predictor variables used in a set of 20 linear mixed-effects models to predict fecal glucocorticoid metabolite (GCM) concentrations, plus the hypothesized effects and explanation of each predictor variable

Fixed effect(s)	Predictor category	Hypothesized mechanism
Plate	Lab	Assay plate error or differences between dates (plates) in laboratory procedures or environment (e.g. temperature, humidity)
Sex	Inter-individual	Sex-associated processes (e.g. pregnancy, lactation, testicular activity)
Day of year (DOY, DOY^2^)	Temporal	Temporal trends in the environment (e.g. snow cover, temperature, plant senescence) or physiology (e.g. reproduction)
Elevation, aspect, site	Broad-scale habitat	Spatial trends in the environment that are generally shared among several territories within a site
Rock size (*L_R1_*, *L_R10_*), mean rock siz e, rock size variation, deepest crevice, graminoid:forb ratio, % forb cover, Graminoid + forb + shrub cover	Fine-scale habitat	Spatial trends in the environment that often vary among territories within a site

Models were fit using the lme4 package (Bates *et al.*, 2015) in R 3.5.1 (R Core Team 2018). We ranked models using Akaike information criterion adjusted for a small sample size (AICc; Burnham and Anderson 2002). Relative support for each focal model was calculated using ΔAICc, the difference in AICc between the focal and ‘top’ (minimum AICc) model, under the assumption that models with ΔAICc > 2 have lower support than the top model (Burnham and Anderson 2002). We also conducted a similar analysis within a generalized linear modeling framework using glmer() and a log link. To improve convergence of parameter estimates and to avoid singular fits, this additional analysis required a set of simplified models with no more than three fixed-effect terms (including any interaction) and one random effect (station or date).

To address our original hypotheses (Fig. 1), if DOY covariates garner high support, then temporally synchronous variation in stress is an important process in our system (Fig. 1a). If DOY covariates are not supported, then the variation in stress is temporally asynchronous or negligible (Fig. 1c). If both DOY and broad or local habitat covariates are supported, then stress varies synchronously over time but differs by territory quality (Fig. 1b). If only broad or local habitat covariates are supported, then differences in territory quality are dominant among the processes we considered (Fig. 1d). Alternatively, if neither habitat covariates nor DOY garner high support, then variations in stress may instead be due to idiosyncrasies among individuals (null model).

## Results

In total, we collected 109 scat samples from 20 stations: 20 samples from 4 stations at CG, 9 samples from 3 stations at LL and 80 samples from 13 stations at WK. The elevation of stations ranged from 3337–3407 m at CG, 3277–3307 m at LL and 3559–3617 m at WK. Three stations at CG faced southwest, and one station faced south; all stations at LL faced northwest; WK had stations at nearly all aspects: two north, two northeast, four east, two southeast, one south, one west and one northwest.

**Figure 3:**
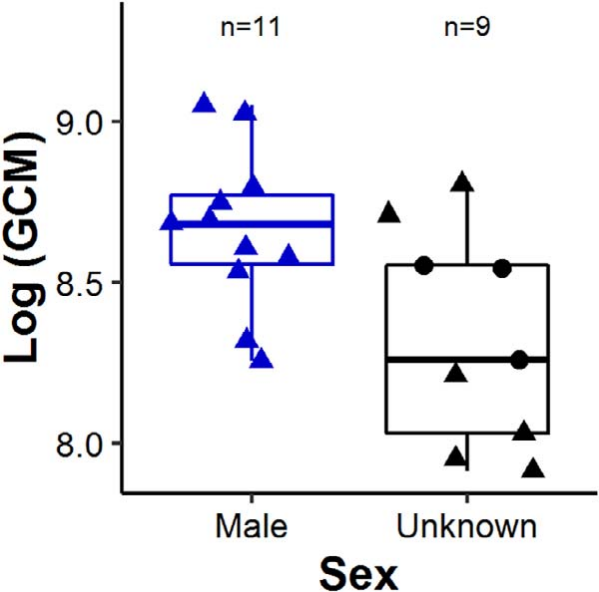
Pika fecal glucocorticoid metabolite (GCM) concentration, log-scaled, by sex. A total of 11 of 20 territory owners were male, while the remaining 9 territory owners were of undetermined sex due to DNA amplification failure (black triangles) or insufficient sample size (black circles). Standard box-and-whisker plots (depicting median, interquartile range and full range excepting outliers), overlaid by actual datapoints (jittered to reduce overlap).

We had sufficient fecal sample size for genetic analyses from 17 of the 20 stations to determine the sex of the territory owner. Genetic analysis of these 17 samples revealed that 11 were deposited by males, and the other six were deemed unknown due to autosomal DNA amplification failure. No females were identified from the available fecal samples. GCM was significantly higher in samples from males than from pikas of unknown sex (Student’s *t*-test, log(GCM), *t* = 3.312, *P* = 0.001; Fig. 3). In model comparisons, we categorized sex either as a two-class variable (*n*(sex) = 11 male, 9 unknown) or a three-class variable (*n*(sex) = 11 male, 6 PCR failure, 3 insufficient sample size).

**Figure 4:**
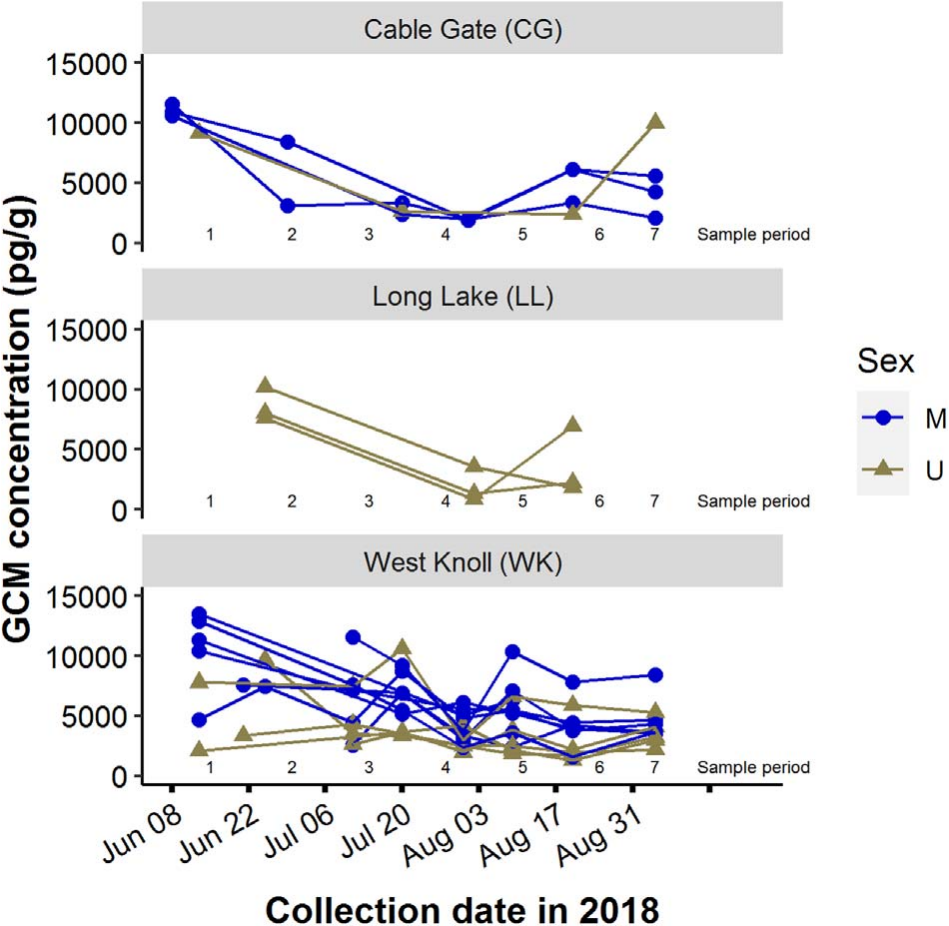
Pika fecal glucocorticoid metabolite (GCM) concentration by collection date in 2018 with sampling period (1–7) superimposed on the x-axis. Each panel displays results from a different site, and line color denotes pika sex.

GCM concentration varied more within stations than among stations. The null model, with only random effects of date and station, revealed that more of the random variance was explained by date (46.64%) than by station (28.74%). A scatterplot also suggests that GCM varied mainly within stations over time rather than among stations (Fig. 4).

**Figure 5:**
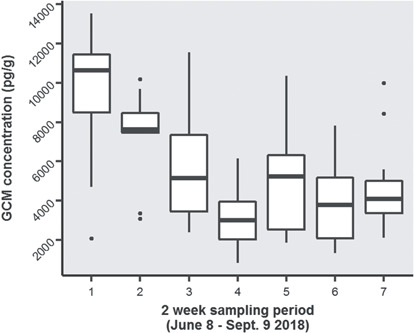
Pika fecal glucocorticoid metabolite (GCM) concentration for all 20 stations grouped by 2-week sampling periods. When available, fecal samples were collected from each station every 2 weeks from 8 June to 9 September 2018. Standard box-and-whisker plots depicting median (heavy horizontal line), interquartile range (box), range (whiskers) and outliers (dots).

To understand temporal trends in stress among individuals, we averaged GCM concentration by 2-week sampling periods. Averaging in this way created seven sampling periods binned every 2 weeks starting 6/8 and ending 9/14. When GCM concentration was averaged across territories, a minimum was observed in sampling period 4 (7/21–8/3, mid-summer; Fig. 5).

The best-supported model of GCM included DOY, DOY^2^ and male sex (Table 3). Similar support was garnered for a model that included DOY^2^, elevation and the interaction between DOY^2^ and elevation. Other well-supported models included terms similar to those in the top model with the addition of one of the broad habitat effects (elevation, site, aspect). Models based on fine scale habitat features (rock size, crevice depth, vegetation characteristics) garnered very low support (ΔAIC > 10). Top models (ΔAIC <  2) were similar between analyses using two- or three-category designations for ‘sex’. Additionally, results using glmer() were similar to those presented here: fitted effect signs were similar among modeling approaches for predictor variables in all supported models, and the best generalized linear models included effects of DOY, DOY^2^ and sex.

**Table 3:** Relative support for models of glucocorticoid metabolite concentration (log-GCM) from the Niwot Ridge LTER. Models are ranked in order of increasing AICc. Only models with ΔAICc < 7 are shown. Top models with equivalent support (ΔAICc < 2) are in bold. The effect sign of each covariate is reported as positive (+), negative (−) or variable (var). Sample size (*n*) was 109 for each model

Ranked models	k	AICc	ΔAICc	Effect sign
**(1) DOY, DOY** ^**2**^ **, male**	7	153.3596	0	
DOY				(−)
DOY^2^				(+)
Male				(+)
**(2) DOY** ^**2**^ **, elevation, DOY** ^**2**^ **: elevation**	7	154.768432	1.4088	
DOY^2^				(+)
Elevation				(+)
DOY^2^ : elevation				(−)
**(3) Elevation, DOY, DOY** ^**2**^ **, male**	8	155.2683	1.9087	
Elevation				(+)
DOY				(−)
DOY^2^				(+)
Male				(+)
(4) Aspect, DOY, DOY^2^, male	8	155.6905	2.3309	
Aspect				(+)
DOY				(−)
DOY^2^				(+)
Male				(+)
(5) DOY^2^, site, DOY^2^ : site	9	156.2011	2.8416	
DOY^2^				(+)
Site				(var)
DOY^2^ : site				(var)
(6) Site, DOY, DOY^2^, male	9	157.1985	3.8390	
Site				(+)
DOY				(−)
DOY^2^				(+)
Male				(+)
(7) DOY, DOY^2^	6	157.5395	4.1799	
DOY				(−)
DOY^2^				(+)
(8) Elevation, DOY, DOY^2^	7	159.0934	5.7338	
Elevation				(+)
DOY				(−)
DOY^2^				(+)
(9) Aspect, DOY, DOY^2^	7	159.7894	6.4298	
Aspect				(+)
DOY				(−)
DOY^2^				(+)

Sampling date and assay plate were somewhat confounded because samples were assayed in chronological order. Thus, we added plate number as a fixed effect to the null model. The plate model ranked 15th among models (ΔAICc = 9.3704), providing little support for a confounding effect of plate. Plate 3 contained samples from mid-season and exhibited most of the lowest GCM concentrations; however, top models were similar when all samples from plate 3 were removed from the analysis.

## Discussion

For highly territorial animals like the American pika, spatial variation in stress may indicate differences in habitat quality among individuals. To understand spatiotemporal trends in stress and the influence of fine- and broad-scale habitat characteristics, we measured the metabolites of stress-associated glucocorticoid hormones in feces (GCMs). In this study, GCMs varied more over time within territories than they varied spatially among territory owners. These results support the hypothesis that individuals experience the same temporally varying stressors, and territories do not differ markedly in quality (Fig. 1a). Although the time series of stress included pairs of individuals that match each of our four hypotheses (e.g. compare Figs 1 and 4), our results may indicate that pikas on Niwot Ridge experience shared physiological and/or climatic drivers of stress, with negligible variation among territories in temporal processes or fine-scale habitat quality.

Relatively low variation in stress among territory owners, compared to variation in stress through time, suggests that territory owners in close proximity might be exposed to similar stressors. Presence of water or ice under talus has a strong correlation with pika presence, persistence and stress (Millar and Westfall 2010; Erb *et al.*, 2011, Wilkening *et al.*, 2015), but on Niwot Ridge, permafrost is unlikely <3700 m (Janke 2005). Our highest station was 3600 m, suggesting that permafrost was absent from all stations. This similarity among habitats might mean that all individuals in this study were exposed to similar seasonal stressors, leading to lower variation in fecal GCM among territories than within territories.

The high temporal variation in stress within stations suggests that pika stressors might vary seasonally. We hypothesize that abiotic factors, such as subsurface talus temperature, and biotic factors, such as reproductive cycles, animal interactions and forage availability, could drive variation. Additionally, our top model supported the fixed effect of DOY (quadratic and linear), suggesting that season is an important driver of stress for pikas in this habitat (Table 3). Specifically, the quadratic DOY term fit the U-shaped curve in average stress throughout the season (Fig. 5). A similar pattern was observed in a smaller study on the Grand Mesa in Colorado, where repeated sampling of scat stations throughout summer 2018 revealed lower average stress in mid-August, compared to earlier and later sampling, suggesting that seasonal stressors may be similar in other habitats (McFarland *et al.*, 2019).

Here, we offer two, non-mutually exclusive hypotheses that might explain higher GCM levels in early summer and fall, relative to mid-summer. First, stress may vary with the seasonality of life history, such as reproduction and dispersal, which might affect pika physiology in a predictable way. Specifically, breeding and reproduction in the spring and early summer might elevate GCM of both sexes. In pikas, a litter is usually conceived 1 month before snowmelt (Millar 1972, Smith 1978), coinciding with our first sampling period. A second period of elevated GCM in the fall may be observed because adult pikas must defend their territories against dispersing juveniles. Second, we hypothesize that stress may vary with the seasonality of forage availability. Specifically, GCM is highest in the beginning of June because winter haypiles are depleted by that time. In mid-June, adult pikas start constructing haypiles (Krear 1965), and through September, 25–55% of their surface activity is spent haying (Dearing 1997). In early fall, pika home range shrinks (Sharp 1973, Tapper 1973) as individuals define their territories, perhaps reducing interactions and movement-related stress. By mid-August in this habitat, most adult pikas have amassed a large haypile (7.7 kg dry weight, Dearing 1997), coinciding with the lowest GCM concentrations (Fig. 5, sampling period 4). GCM concentrations may then increase again as pikas defend these haypiles.

Two models with high support suggest elevation is an important covariate of stress in our system (Table 3). In particular, the negative interaction of DOY^2^ and elevation suggests that the strength of the u-shaped effect of DOY (Fig. 5) is more pronounced in territories at lower elevations (<3500 m) (see Supplementary Fig. 1). We used elevation as a proxy for broad spatial trends in the environment that are generally shared among several territories within a site. We hypothesize that habitat and territory quality may be less variable at lower elevations compared to higher elevations, thereby making the seasonal pattern (i.e. the pronounced u-shaped curve) more detectable. The stronger seasonal signal at lower elevations suggests climatic forcing, rather than seasonal patterns in physiology, has more of an effect on our metric of stress. All high elevation territories (>3500 m) were located within one site, WK, thereby confounding elevation with site. WK is unique from the other sites in that it is located above treeline, it is exposed to high winds from the west, and persistent snow accumulates on the east-facing slope of the knoll. These local idiosyncrasies may also partially explain the higher variation in our stress metric. However, territories at all sites share similar trends in stress levels through time (Fig. 4). This shared seasonal trend among sites could reflect shared weather patterns experienced in the region during the year of our study, rather than site-specific patterns in temperature, snowmelt or other processes such as predator dynamics. Future studies that explicitly consider temperature and/or local microclimates could clarify the effect of weather patterns on stress. For example, a study of pikas in Washington found that corticosterone levels detected in summer coat hair were influenced by lower mean maximum daily temperatures, providing preliminary evidence that cold temperatures experienced in June to mid-July can cause thermal stress (Waterhouse *et al.*, 2017). Finally, temperature (subsurface and ambient) is a predictor of pika occupancy and abundance in many systems (Hafner 1994; Beever *et al.*, 2010; Wilkening *et al.*, 2011, 2015; Yandow *et al.*, 2015; Schwalm *et al.*, 2016; Wright and Stewart 2018), further highlighting the value of future studies that explicitly investigate the effects of temperature on seasonal patterns of stress.

GCM levels in our study were within the range of levels found in other studies of pikas in this and other habitats (2177–15 800 pg/g; Wilkening *et al.*, 2013, 2015, 2016; Wilkening and Ray 2016). Additionally, our results are consistent with findings in other small mammals. Average GCM concentration in the current study was 5176 pg/g, similar to the baseline fecal GCM levels reported in North American red squirrels (*Tamiasciurus hudsonicus*; 6040 pg/g, Dantzer *et al.*, 2010). Our findings that GCM was highest in the spring and decreased as the summer progressed also mirror findings for arctic ground squirrels (*Urocitellus parryii*, Sheriff *et al.*, 2012), yellow-bellied marmots (*Marmota flaviventris*, Smith *et al.*, 2012) and chipmunks (*Tamias speciosus*, Hammond *et al.*, 2018). This trend could be attributed to the shared timing of reproduction early in the year for these species or to shared seasonal stressors in alpine environments.

Additionally, it is important to consider confounding effects of environmental conditions after fecal deposition (Washburn and Millspaugh 2002, Millspaugh and Washburn 2004, Stetz *et al.*, 2013, Dantzer *et al.*, 2014, Wilkening *et al.*, 2016, Lafferty *et al.*, 2019). A recent study on snowshoe hares (*Lepus americanus*) found post-deposition effects of temperature and precipitation on fecal GCM concentrations (Lafferty *et al.*, 2019). Specifically, snowshoe hare feces exposed to cool and dry (vs. warm and/or wet) climates showed the least variation in GCM concentration over a 6-day period (Lafferty *et al.*, 2019). Likewise, a study on pikas found confounding effects of temperature and precipitation, but samples experimentally exposed to conditions in similar ecoregions were not significantly affected by post-deposition effects (Wilkening *et al.*, 2016). Our study took place in a relatively small area in the southern Rocky Mountains that experiences relatively cool and dry summers, and our stations were in close proximity (max distance between stations <2.85 km, Fig. 1). To minimize post-deposition effects, we collected fresh pellets every 2 weeks (the minimum time required to accumulate a sufficient number of pellets for GCM analysis), cleared stations between sampling events and transferred samples to a freezer within 24 hours of collection.

Sex determination was always based on sub-samples from one time point during the study. Therefore, it is possible that the territory owner (and its sex) changed during the sampling season, which could have affected seasonal variation in stress. Occupancy turnover rates at the scale of territories are highly dependent on local influences (i.e. climatic patterns or habitat quality) but can be as high as 50% between years in some populations (Rodhouse *et al.*, 2018). Genetic analysis revealed that the majority of territory owners within our study was male. However, six samples were designated ‘unknown’ due to repeated lack of amplification (Fig. 3), likely because samples did not contain enough genetic material. In the same PCR analyses, pika feces from other studies were successfully identified as originating from females (A. A. Hove, unpublished data). Although it is possible that females could be present in our six unknown samples, it is also possible that males are more territorial (Krear 1965) and/or more likely to mark their territories with conspicuous piles of fecal pellets. We offer this hypothesis as an explanation for the apparent male bias in our sampling. Although sex-specific differences in territorial behaviors are not well documented in pikas, in another wild lagomorph (the European rabbit, *Oryctolagus cuniculus*), adult males visit latrines more frequently than adult females (Sneddon 1991), increasing the likelihood of a male-dominant latrine. Finally, although perhaps unlikely, it is also possible that, because males and females usually alternate territories (Krear 1965, Sharp 1973, Tapper 1973, Smith and Ivins 1984, Brandt 1989), the spacing of our stations (>50 m) may have systematically skipped over females.

Our results suggest that individuals within a population can respond synchronously to abiotic or biotic stressors, and therefore that climatic stressors should be considered as a potential threat to pika populations. Synchronous responses to extreme events caused by climate change (e.g. reduced snowpack, hot temperatures, change in forage, etc.) could cause whole populations to be lost at one time. Such responses are likely exacerbated by the species’ limited dispersal capabilities (Smith 1974b; Smith and Ivins 1983; Peacock 1997; Castillo *et al.*, 2014, 2016) and could therefore put pikas at risk of local extirpation (Beever *et al.*, 2011, 2016; Calkins *et al.*, 2012; Nichols *et al.*, 2016; Stewart *et al.*, 2017). Although stress is known to vary seasonally in some systems (Romero 2002, Baker *et al.*, 2013, Dantzer *et al.*, 2014), our study is among the few that have recorded temporal variation in a stress metric from individual territories of wild animals. If conducted over several years, similar studies could address whether individual stress levels within a population vary with interannual differences in climatic factors. Such replication could potentially disentangle shared seasonal climatic drivers from seasonal physiological changes or the weather of one particular season.

Although we observed more temporal variation than spatial variation in our stress metric, other studies should be conducted within more heterogeneous landscapes and at larger spatial scales to determine whether stress levels respond more to shared seasonal trends or fine-scale habitat differences. Further studies will also help determine whether non-invasive analyses of fecal pellets could provide insights on the relative quality of pika territories across a more heterogeneous landscape. Using non-invasive sampling techniques complicated definitive identification of individuals in our study, reducing our ability to analyse effects of individual level characteristics. However, the methods we used for investigating stress at the population level are accessible to managers and could be integrated into established pika monitoring efforts to provide physiological data for populations of concern. Using GCM or other stress indices as a metric of individual health within a population has been suggested as a way for managers to predict population declines before they occur, thus identifying situations where management interventions (e.g. translocation or habitat modifications) may be warranted (Bergman *et al.*, 2019). Our results specifically suggest the importance of considering sampling date such that seasonal patterns in GCM are not mistaken for true baseline variation in stress among populations. The relative effects of habitat quality, climate and life history on stress are understudied topics in conservation physiology (Homyack 2010; Baker *et al.*, 2013) that hold promise for a more mechanistic understanding of population health.

## Supplementary Material

supplementary_Fig1_tiff_coab024
