# Supplementary figures and images for: Temporal vs. spatial variation in stress-associated metabolites within a population of climate-sensitive small mammals
Source: Conserv Physiol. 2021 May 3;9(1):coab024. doi: 10.1093/conphys/coab024 (PMC8127223; doi:10.1093/conphys/coab024)

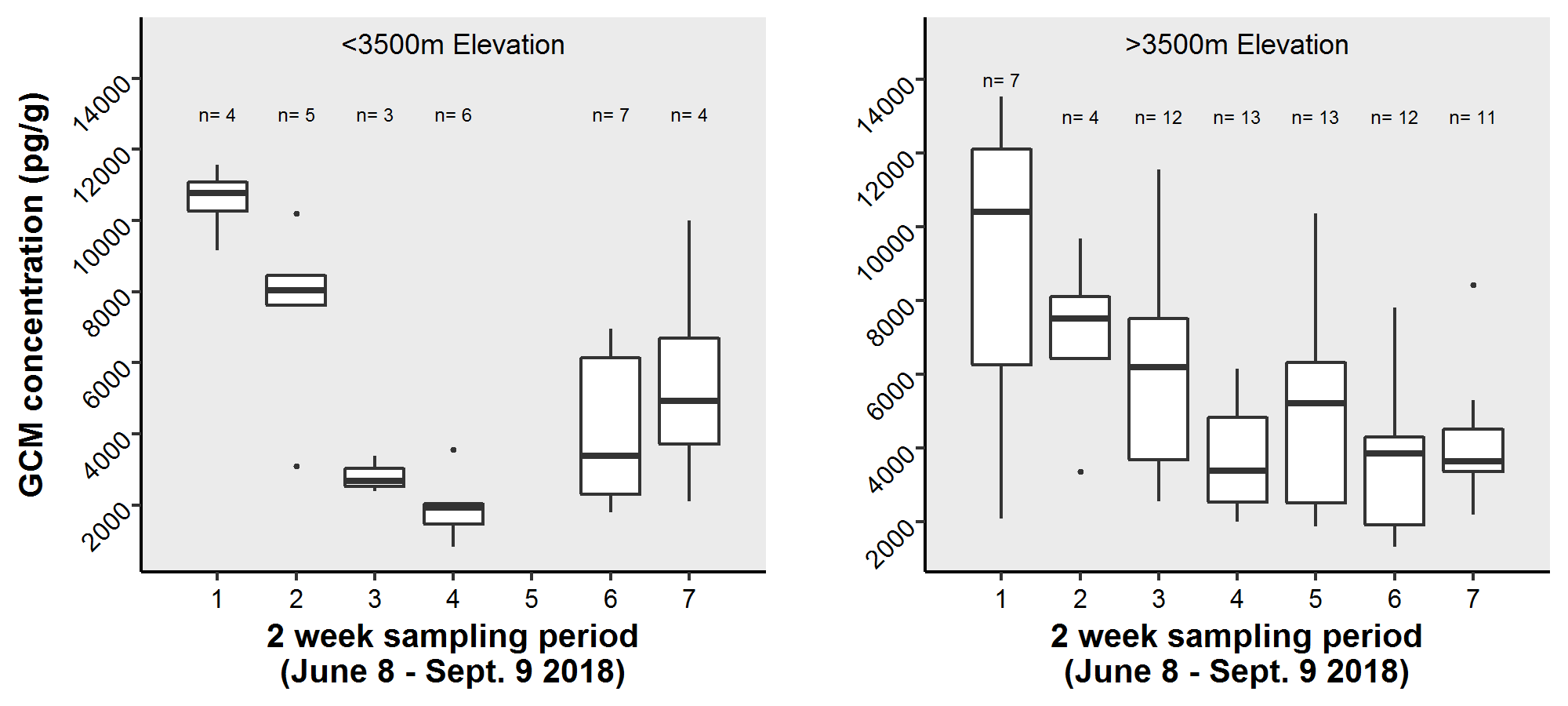

Supplement: supplementary_Fig1_tiff_coab024 [file supplementary_fig1_tiff_coab024.png]
